# Low Body Mass Index as a Predictor of Amiodarone‐Induced Pulmonary Toxicity

**DOI:** 10.1002/joa3.70205

**Published:** 2025-10-22

**Authors:** Takuto Zaizen, Hidekazu Kondo, Teruo Noguchi, Reina Tonegawa‐Kuji, Tsukasa Kamakura, Mitsuru Wada, Kohei Ishibashi, Yuko Inoue, Koji Miyamoto, Takeshi Aiba, Naohiko Takahashi, Kengo Kusano

**Affiliations:** ^1^ Department of Cardiology and Clinical Examination, Faculty of Medicine Oita University Yufu‐City Oita Japan; ^2^ Department of Cardiovascular Medicine National Cerebral and Cardiovascular Center Suita Osaka Japan; ^3^ Department of Medical and Health Information Management National Cerebral and Cardiovascular Center Suita Osaka Japan

**Keywords:** amiodarone, body mass index, pulmonary toxity, risk factor

## Abstract

**Background:**

Amiodarone‐induced pulmonary toxicity (APT) is one of the major side effects of the medication when used in the treatment of arrhythmia. However, the risk factors for developing APT have yet to be fully understood.

**Methods and Results:**

We retrospectively analyzed 454 patients who were treated with amiodarone for arrhythmia between 2016 and 2020 at the National Cerebral and Cardiovascular Center, Osaka, Japan. During the median follow‐up period of 207 days, 24 patients (5.4%) had APT. Using a multivariate analysis of the Cox proportional hazards model, lower body mass index (BMI) (hazard ratio [HR]: 0.81, 95% confidence interval [CI]: 0.71–0.95), higher age (HR: 1.06, 95% CI: 1.02–1.10), and higher amiodarone maintenance dose (HR: 1.01, 95% CI: 1.003–1.02) were risk factors for APT. Specifically, the patients whose BMIs were < 22 kg/m^2^ were approximately three times more likely to develop APT than the patient whose BMIs were ≥ 22 kg/m^2^. The cutoff value for maximum KL‐6 levels during amiodarone therapy as an APT screening test was 444 U/mL or higher, with a sensitivity of 70.8% and specificity of 88.1%.

**Conclusion:**

Lower BMI, higher age, and a higher maintenance dose were identified as independent risk factors for APT. KL‐6 levels during administration may be useful in suspecting the development of APT.

## Graphical abstract

This retrospective study analyzing 454 patients who were treated with amiodarone revealed that the patients whose BMIs were < 22 kg/m^2^ were more likely to develop amiodarone‐induced pulmonary toxicity. The cutoff value for maximum KL‐6 levels during amiodarone therapy as an APT screening test was 444 U/mL.

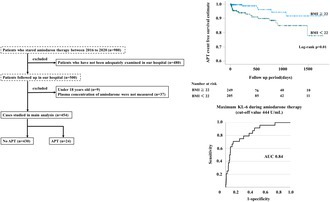

## 1 Introduction

Amiodarone is an antiarrhythmic agent that has been widely used in the treatment of ventricular and supraventricular arrhythmias [1]. Due to its high lipophilicity, amiodarone has various extracardiac side effects, including thyroid dysfunction, pulmonary toxicity, hepatotoxicity, and dermatitis [2]. Particularly, amiodarone‐induced pulmonary toxicity (APT) can have fatal consequences.

Previous studies have reported that the incidence of APT was 1%–10% [3, 4, 5, 6, 7] and the mortality rate was 10%–37% [8, 9, 10]. When amiodarone was first developed, a high maintenance dose (400 mg) was typically administered. However, because high‐dose amiodarone was reported to often cause APT [7, 11], the recommended dose was reduced. Despite this change, even low‐dose amiodarone, such as doses less than 200 mg, still causes APT [12].

Beyond dosing, it is important to identify the risk factors of APT. The currently known major risk factors include greater age, pre‐existing lung diseases, such as chronic pulmonary obstructive pulmonary disease (COPD), male gender, and renal diseases [3, 4, 13].

KL‐6 is a high‐molecular‐weight mucin‐like glycoprotein secreted by proliferating type II alveolar lung cells and is a sensitive marker of disease activity in various interstitial lung diseases [14]. Elevated serum KL‐6 levels have also been reported in patients with APT [15]; however, their sensitivity and specificity as a screening test for APT have not yet been established.

The aim of this study is to reinvestigate the risk factors for the development of APT and the diagnostic performance of KL‐6 as a potential screening test in patients who were treated with amiodarone in a large cardiovascular center in Japan.

## 2 Methods

This study was approved by the institutional review board of the National Cerebral and Cardiovascular Center. Since this is a retrospective study, an opt‐out method was allowed. The details of the research were disclosed in advance on the National Cerebral and Cardiovascular Center's website.

**FIGURE 1:**
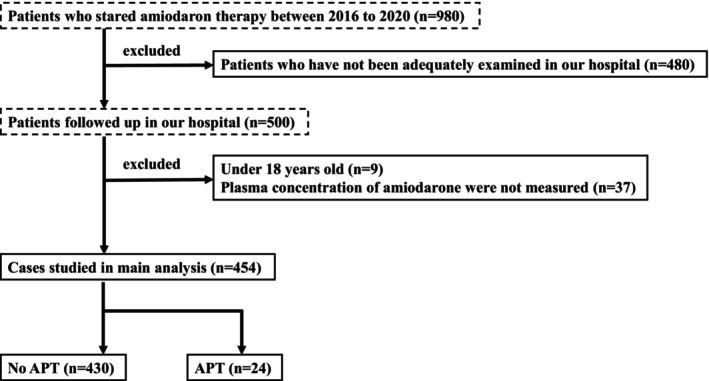
Flowchart of the study. APT, amiodarone induced pulmonary toxicity.

To identify risk factors for APT, we retrospectively investigated the clinical courses of patients who were taking oral amiodarone for arrhythmia. We reviewed the records of 980 patients who started oral amiodarone at the National Cardiovascular Center between January 2016 and December 2020 (Figure 1). Follow‐up data were collected until December 2022. The following patients were excluded: 480 patients who had not been adequately examined (patients with inadequate examination were defined as those who had not undergone blood tests or echocardiography in our hospital prior to the start of amiodarone therapy and/or who did not have information on their height and weight at the start of amiodarone therapy), nine children under 18 years of age, and 37 patients whose plasma concentrations of amiodarone or monodesethylamiodarone were not measured. Finally, 454 patients were included in the study. In the analysis to assess the accuracy of serum KL‐6 levels as a screening test for APT, 191 patients who lacked data on serum KL‐6 levels were excluded (Figure S1).

Based on the previous study [4], APT was diagnosed when the following criteria were met: (1) New onset of pulmonary symptoms such as dyspnea, cough, pleuritic chest pain, and fever; (2) new chest abnormalities detected by chest X‐ray and computed tomography (CT); and (3) no evidence supporting congestive heart failure, infectious processes, or malignancy.

Medical records were reviewed and the following data were collected at the start of amiodarone administration: age, gender, body mass index (BMI), type of arrhythmia that warranted amiodarone treatment, underlying cardiac disease, loading and maintenance dose of amiodarone, left ventricular ejection fraction (LVEF) measured by echocardiography, smoking history, history of respiratory diseases (e.g., COPD and bronchial asthma), and other concomitant diseases (e.g., hypertension, dyslipidemia, diabetes, congestive heart failure). The plasma concentrations of amiodarone and its metabolites were measured at the last visit after the maintenance dose was determined.

## 3 Statistics

The cumulative incidence of APT was estimated using the Kaplan–Meier method from the start of drug administration to the last observed day or the day diagnosed with APT. The incidence and mortality rate of APT was calculated in 100 person‐years method. We used the Mann–Whitney test to compare quantitative data and a *χ* ^2^ test to compare categorical variables.

To identify risk factors for the development of APT, a multivariate analysis using the Cox proportional hazards model was performed using data from the start of amiodarone administration. Variables which were significant in univariate analysis were selected as independent variables for multivariate analysis. Both CKD and eGFR were significant in the univariate analysis; however, because of their correlation, only eGFR was included as a covariate in the multivariate analysis. Although the WHO defines a normal BMI as 18.5–25 kg/m^2^, the Japan Society for the Study of Obesity defines a BMI of 22 kg/m^2^ as the standard. Therefore, we compared the risk of developing APT between two groups: patients whose BMIs were < 22 kg/m^2^ and patients whose BMIs were ≥ 22 kg/m^2^. Differences in the incidence of APT between the two groups were compared using the Kaplan–Meier method and the log‐rank test. Using the Youden index method based on the receiver‐operating characteristic (ROC) curve, we determined the cutoff value of low BMI as a risk factor for the development of APT. Because a lower BMI was associated with a higher risk of APT, the ROC curve was constructed using inverted BMI values.

A logistic analysis was used to examine the association between plasma concentrations of amiodarone and its metabolites and the development of APT. This analysis was performed separately from the Cox proportional hazards model that explored risk factors at the time of amiodarone initiation, because the point in time at which the variables were measured was different. Age, BMI, and the maintenance dose of amiodarone, which were significant in the Cox proportional hazards model, were also included as variables.

To evaluate the accuracy of the serum KL‐6 value as a screening test for APT, sensitivity and specificity were calculated using receiver‐operating characteristic (ROC) curve. The cutoff value was determined by the Youden index, which is the point where the sum of sensitivity and specificity is maximized. ROC curve was performed by complete case analysis, excluding patients who have missing data for KL‐6 (*n* = 191). Serum KL‐6 values were adopted as the maximum values during amiodarone treatment. A *p* value of less than 0.05 was considered statistically significant.

## 4 Results

### 4.1 Incidence and Prognosis of APT

**FIGURE 2:**
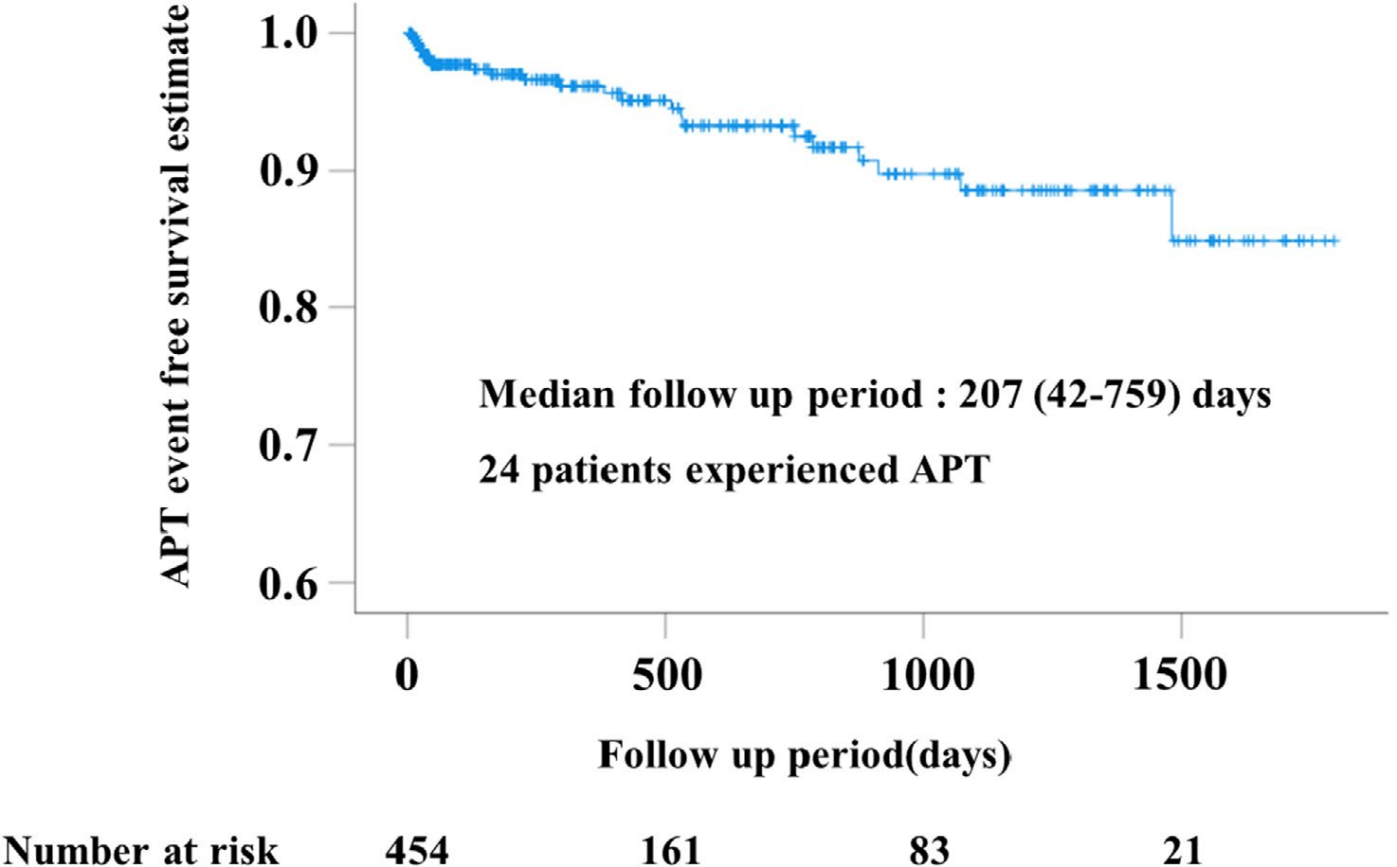
Kaplan–Meier curves for freedom from APT. APT, amiodarone induced pulmonary toxicity.

The median follow‐up period was 207 [42–759] days, and 24 patients (5.3%) were diagnosed with APT. The cumulative incidence of APT is presented using the Kaplan–Meier method (Figure 2). The incidence rate of APT was 4.2 per 100 person‐years. Five of the 24 patients (20.8%) died from APT. The median time from the onset of APT to death was 34 [18–108] days, and the mortality rate was 12.4 per 100 person‐years.

### 4.2 Clinical Characteristics of the Study Participant

Table 1 shows clinical characteristics of the study participants (All patients, No APT group, and APT group). In all patients, the median age was 68 [55–76] years old, 310 of 454 (68.3%) were male, and the median BMI was 22.4 [20.1–25.1] kg/m^2^. Amiodarone was more commonly used in patients with reduced ejection fraction, with a median LVEF of 35%. The most common underlying cardiac disease was non‐ischemic cardiomyopathy, and the most common arrhythmia was atrial fibrillation (AF) and atrial flutter (AFL). The median loading dose of amiodarone was 200 [100–200] mg, and the median maintenance dose was 100 [50–100] mg. The median plasma concentrations of amiodarone and monodesethylamiodarone were 0.50 [0.33–0.77] μg/mL and 0.34 [0.21–0.50] μg/mL, respectively. In comparison with the Non‐APT group and APT group, the APT group was significantly older, had a lower BMI, and higher plasma concentrations of amiodarone and monodesethylamiodarone than the group without APT.

**TABLE 1:** Baseline characteristics of the study participants.

	All (*n* = 454)	No APT (*n* = 430)	APT (*n* = 24)	*p*
Age, years	68 (55–76)	68 (54–76)	72 (68–80)	0.011
Male, *n*	310 (68.3%)	291 (67.7%)	19 (79.2%)	0.24
BMI, kg/m^2^	22.4 (20.1–25.1)	22.4 (20.1–25.2)	20.2 (19.0–21.9)	0.005
LVEF, %	35 (24–55)	35 (24–55)	31 (24–43)	0.49
Congestive heart failure, *n*	354 (78.0%)	332 (77.2%)	22 (91.7%)	0.096
Underlying heart disease, *n*				0.20
Ischemic heart disease	136 (30.0%)	126 (29.3%)	10 (41.7%)	
Non‐ischmic cardiomyopathies	217 (47.8%)	208 (47.1%)	9 (37.5%)	
Valvular disease	43 (9.5%)	39 (9.1%)	4 (16.7%)	
Others	58 (12.8%)	57 (13.3%)	1 (4.2%)	
Arrhythmia, *n*				0.47
VT/VF	151 (33.3%)	143 (33.3%)	8 (33.3%)	
NSVT/PVC	71 (15.6%)	69 (16.0%)	2 (8.3%)	
AF/AFL	193 (42.5%)	182 (42.3%)	11 (45.8%)	
SVT/PAC	39 (8.6%)	36 (8.4%)	3 (12.5%)	
Smoking history, *n*	258 (57.8%)	242 (57.1%)	16 (72.7%)	0.15
Respiratory disease, *n*	18 (4.0%)	16 (3.7%)	2 (8.3%)	0.26
Chronic kidney disease, *n*	284 (62.6%)	265 (61.6%)	19 (79.2%)	0.084
Chronic liver disease, *n*	26 (5.7%)	26 (6.0%)	0 (0.0%)	0.22
Hypertention, *n*	318 (70.0%)	302 (70.2%)	16 (66.7%)	0.71
Dyslipidemia, *n*	277 (61.0%)	262 (60.9%)	15 (62.5%)	0.88
Diabetes mellitus, *n*	68 (15.0%)	64 (14.9%)	4 (16.7%)	0.81
AMD loading dose, mg	200 (100–200)	200 (100–200)	200 (100–275)	0.25
AMD maintenance dose, mg	100 (50–100)	100 (50–100)	100 (50–200)	0.95
Plasma concentration of AMD, μg/mL	0.50 (0.33–0.77)	0.48 (0.33–0.76)	0.69 (0.55–0.90)	0.007
Plasma concentration of MDEA, μg/mL	0.34 (0.21–0.50)	0.34 (0.21–0.49)	0.45 (0.28–0.57)	0.092
ACEi or ARB, *n*	293 (64.5%)	279 (67.9%)	14 (58.3%)	0.51
β blocker, *n*	326 (71.8%)	307 (71.4%)	19 (79.2%)	0.41
Diuretic drug, *n*	305 (67.2%)	289 (67.2%)	16 (66.7%)	0.96
Statin, *n*	200 (44.1%)	188 (43.7%)	12 (50.0%)	0.55

*Note:* Date are Median (25th percentile–75th percentile) or number (%).

Abbreviations: ACEi, angiotensin converting enzyme inhibitor; AF, atrial fibrillation; AFL, atrial flutter; AMD, amiodarone; ARB, angiotensin2 receptor blocker; BMI, body mass index; LVEF, left ventricular ejection fraction; MDEA, monodesethylamiodarone; NSVT, non‐sustained ventricular tachycardia; PAC, premature atrial contraction; PVC, premature ventricular contraction; SVT, supraventricular tachycardia; VF, ventricular fibrillation; VT, ventricular tachycardia.

### 4.3 Risk Factors of APT

**FIGURE 3:**
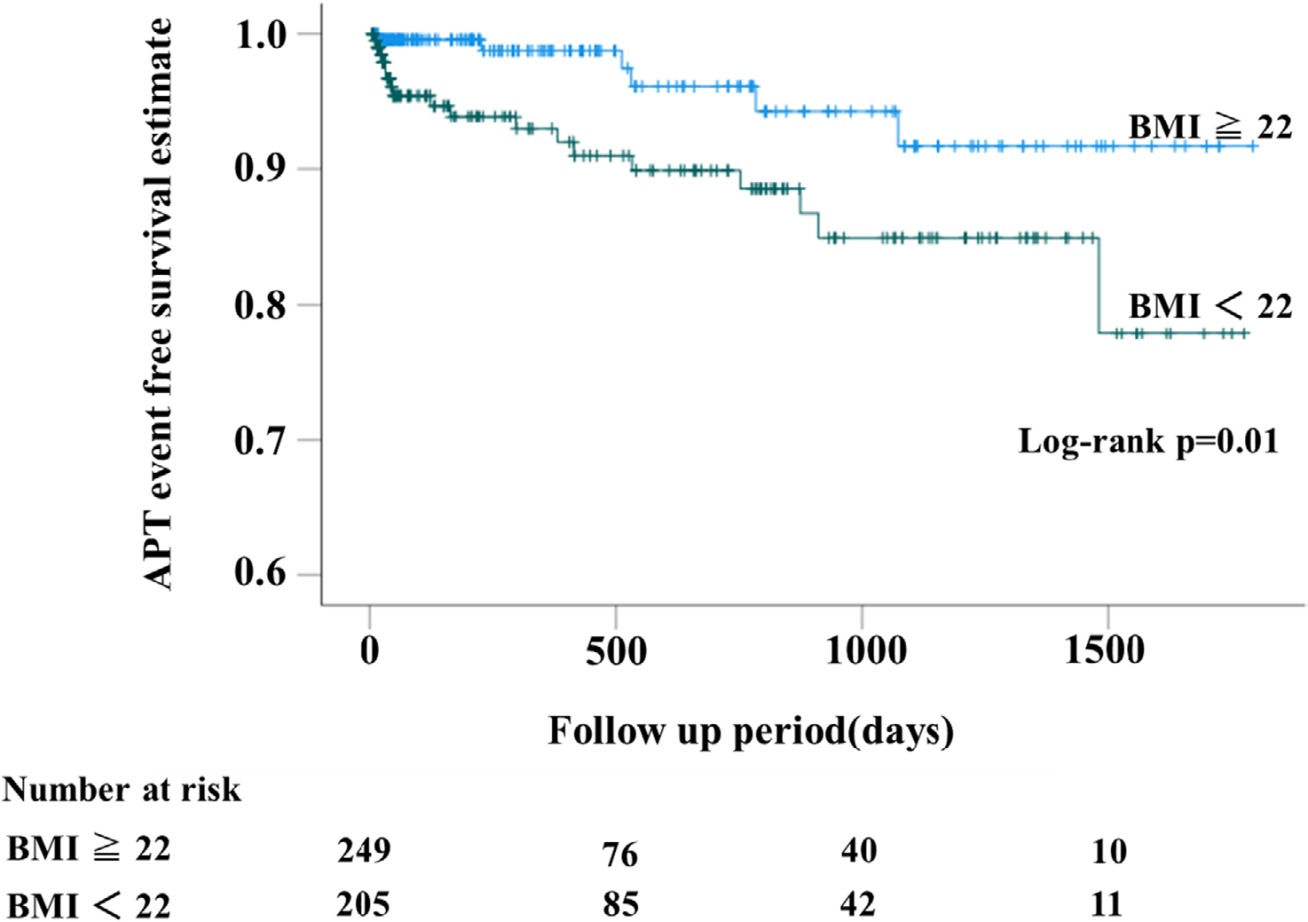
Kaplan–Meier curves for freedom from APT in patients with BMI ≥ 22 or BMI < 22. APT, amiodarone induced pulmonary toxicity; BMI, body mass index.

Using a multivariate analysis of the Cox proportional hazards model, lower BMI, higher age, and higher amiodarone maintenance dose were risk factors for APT at the start of amiodarone therapy (Table 2). The hazard ratio was 1.06 (95% confidence interval [CI] 1.02–1.10) for age, 0.81 (95% CI 0.71–0.95) for BMI, and 1.01 (95% CI 1.003–1.02) for amiodarone maintenance dose. When the risk of developing APT was compared using the Kaplan–Meier method for the two groups whose BMIs were either < 22 kg/m^2^ or ≥ 22 kg/m^2^, the risk of developing APT was significantly higher in the BMI < 22 kg/m^2^ group (*p* = 0.01) (Figure 3). The hazard ratio of developing APT for BMI < 22 kg/m^2^ was 3.11 (95% CI 1.22–7.91) by multivariate Cox proportional model (Table 3). The cutoff value of BMI for predicting APT, as determined by ROC analysis, was ≤ 21.5 kg/m^2^, with a sensitivity of 75%, specificity of 59.5%, positive predictive value of 9.4%, negative predictive value of 97.7%, and an area under the curve (AUC) of 0.67 (Figure S2).

**TABLE 2:** Univariate and multivariate Cox proportional hazards model for prediction of APT at the start of treatment.

	Univariate analysis		Multivariate analysis	
	Hazard ratio (95% CI)	*p*	Hazard ratio (95% CI)	*p*
Age, years	1.06 (1.02–1.10)	0.002	1.06 (1.02–1.10)	0.006
Male, *n*	0.50 (0.18–1.33)	0.16		
BMI, kg/m^2^	0.86 (0.76–0.98)	0.018	0.81 (0.71–0.95)	0.009
LVEF, %	0.99 (0.96–1.01)	0.35		
Congestive heart failure, *n*	3.30 (0.77–14.02)	0.11		
Ischemic heart disease	1.94 (0.86–4.37)	0.11		
Ventricular arrhythmia	0.66 (0.29–1.50)	0.32		
Smoking history, *n*	2.18 (0.85–5.56)	0.11		
Respiratory disease, *n*	2.76 (0.65–11.76)	0.17		
Chronic kidney disease, *n*	2.81 (1.05–7.54)	0.040		
Hypertention, *n*	0.75 (0.10–5.53)	0.78		
Dyslipidemia, *n*	0.81 (0.11–6.0)	0.83		
Diabetes mellitus, *n*	1.6 (0.54–4.73)	0.39		
BNP, pg/mL	1.0 (1.0–1.001)	0.41		
eGFR, mL/min/1.73m^2^	0.98 (0.96–0.997)	0.025	0.995 (0.98–1.02)	0.61
T‐Bil, mg/dL	1.04 (0.84–1.28)	0.73		
AMD loading dose, mg	1.003 (0.999–1.01)	0.16		
AMD maintenance dose, mg	1.01 (1.002–1.02)	0.014	1.01 (1.003–1.02)	0.004
ACEi or ARB, *n*	0.79 (0.36–1.71)	0.55		
β blocker, *n*	1.20 (0.45–3.21)	0.72		
Diuretic drug, *n*	0.96 (0.41–2.24)	0.92		
Statin, *n*	1.40 (0.63–3.11)	0.42		

Abbreviations: ACEi, angiotensin converting enzyme inhibitor; AMD, amiodarone; ARB, angiotensin2 receptor blocker; BMI, body mass index; BNP, brain natriuretic peptide; CI, confidence interval; eGFR, estimated glomerular filtration rate; LVEF, left ventricular ejection fraction; MDEA, monodesethylamiodarone; T‐Bil, total bilirubin.

**TABLE 3:** Univariate and multivariate Cox proportional hazards model for prediction of APT in patients with BMI < 22 kg/m^2^ at the start of treatment.

	Univariate analysis		Multivariate analysis	
	Hazard ratio (95% CI)	*p*	Hazard ratio (95% CI)	*p*
BMI < 22, kg/m^2^	3.22 (1.28–8.14)	0.013	3.11 (1.22–7.91)	0.017
Age, years	1.06 (1.02–1.10)	0.002	1.06 (1.02–1.10)	0.004
AMD maintenance dose, mg	1.01 (1.002–1.02)	0.014	1.01 (1.003–1.10)	0.003

Abbreviations: AMD, amiodarone; BMI, body mass index; CI, confidence interval.

Logistic analyses were performed to investigate the association between plasma concentrations of amiodarone and monodestylamiodarone and the development of APT, but none of the differences was significant (Table 4).

**TABLE 4:** Univariate and multivariate logistic regression analysis for prediction of APT.

	Univariate analysis		Multivariate analysis	
	Odds ratio (95% CI)	*p*	Odds ratio (95% CI)	*p*
Age, years	1.05 (1.01–1.09)	0.01	1.05 (1.01–1.09)	0.008
BMI, kg/m^2^	0.85 (0.75–0.96)	0.009	0.83 (0.72–0.96)	0.01
AMD maintenance dose, mg	1.002 (0.996–1.008)	0.48		
Plasma concentration of AMD, μg/mL	2.14 (0.98–4.67)	0.057	2.06 (0.79–5.37)	0.14
Plasma concentration of MDEA, μg/mL	3.15 (0.61–16.20)	0.17	1.65 (0.27–10.05)	0.59

Abbreviations: AMD, amiodarone; BMI, body mass index; CI, confidence interval; MDEA, monodesethylamiodarone.

### 4.4 APT Screening Test

**FIGURE 4:**
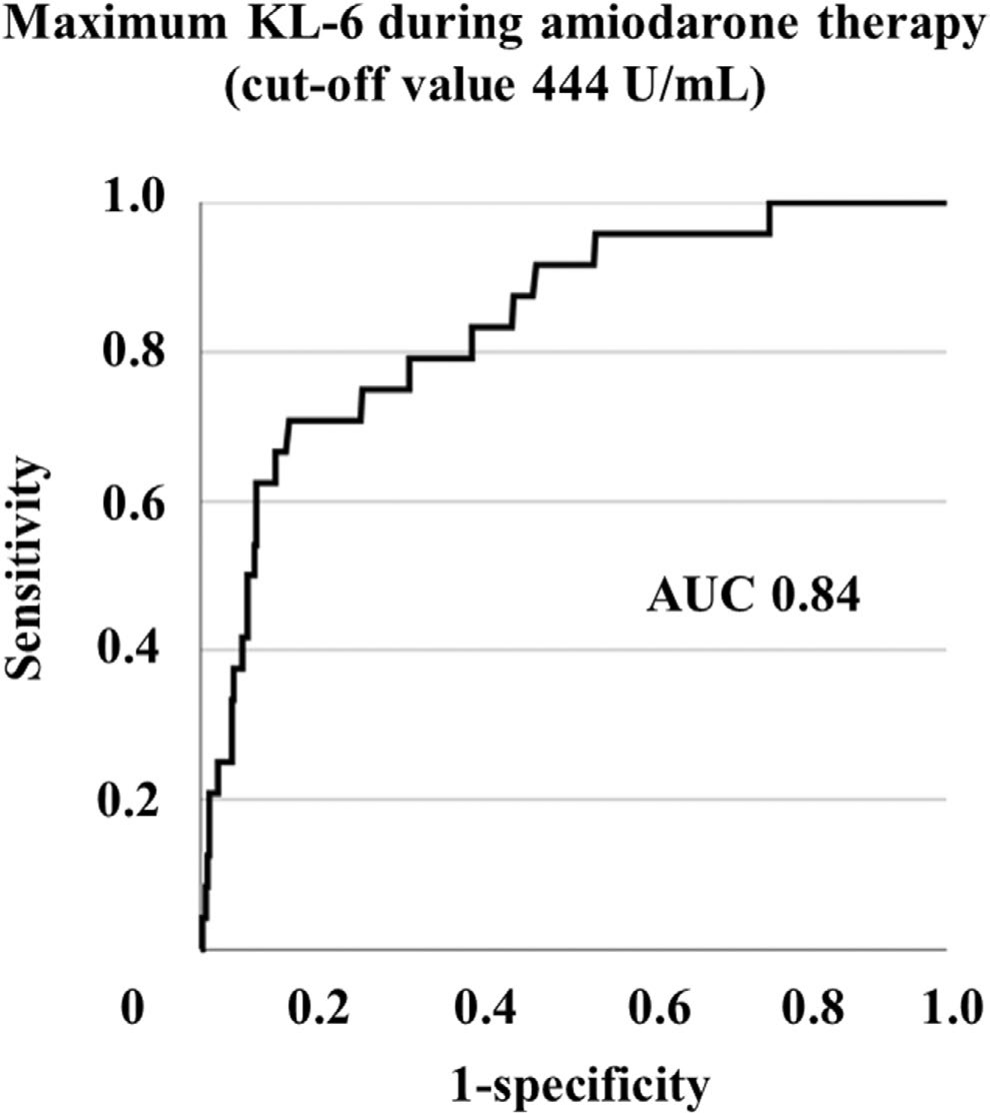
Receiver‐operating characteristic plot for percent predicted serum KL‐6 as screening tests of pulmonary toxicity during amiodarone therapy. AUC, area under the curve.

The ROC curve of the APT screening test by KL‐6 levels is shown in Figure 4. The area under the curve (AUC) of the ROC curve was 0.84. The cutoff value for the maximum KL‐6 levels during amiodarone therapy was 444 U/mL or higher, with a sensitivity of 70.8% and specificity of 88.1%.

## 5 Discussion

This study was a retrospective observational study examining risk factors for APT. During the median follow‐up of 207 days, the incidence of APT was 5.3%. Lower BMI, older age, and higher amiodarone maintenance dose were independent risk factors for developing APT. Specifically, the patients whose BMIs were < 22 kg/m^2^ were approximately three times more likely to develop APT than the patients whose BMIs were ≥ 22 kg/m^2^. Plasma concentrations of amiodarone and monodesethylamiodarone were not significant as risk factors.

A recent multicenter retrospective cohort study in Hong Kong reported that APT occurred in 34 of 1786 (1.9%) patients [13]. The mean age of the patients with APT was 78.6 ± 11.7 years and without APT was 73.6 ± 14.1 years. The mean duration of amiodarone treatment was 70.5 ± 52.4 months, and the mean amiodarone maintenance dose was 170.6 ± 52.4 mg. Though they concluded that higher age, ventricular arrhythmia, underlying pulmonary disease, and cumulative dose of amiodarone were risk factors for developing APT, BMI was not examined. A retrospective observational study in Japanese patients reported that APT occurred in 40 of 500 (8.0%) patients [4]. The mean age was 53 ± 16 years, the mean follow‐up period was 48 ± 43 months, and the mean maintenance dose of amiodarone was 141 ± 70 mg. Risk factors for developing APT were older age, higher maintenance dose, and higher plasma concentration of monodesethylamiodarone, while BMI was comparable between the two groups.

Consistent with these previous studies, an older age and a higher amiodarone maintenance dose were significant risk factors for developing APT, while lower BMI was found to be a new significant risk factor in the present study. In comparison to a retrospective observational study in Japan, our study may have been influenced by the relatively older age of the subjects. However, compared with the Hong Kong report, the APT incidence was higher in the present study despite the younger age of the subjects. This may be due to the racial and BMI differences. This study is the first to report BMI as a risk factor in APT in which we have identified that a lower BMI is a potential risk factor for developing APT.

Amiodarone has a large volume of distribution and its pharmacokinetics are diverse. It is highly liposoluble [16] where it preferentially distributes in adipose tissue, lung, thyroid gland, kidney, and liver, in that order. The metabolite has the highest affinity for lungs followed by kidney, thyroid gland, adipose tissue, and liver [17]. Though the mechanism by which amiodarone causes various organ damage has not been clearly elucidated, amiodarone and its metabolites cause extracardiac side effects of the organs where they accumulate in.

Lower BMI has not been reported as a risk factor for APT but has been reported as a risk factor for amiodarone‐induced thyroid dysfunction [18]. Because amiodarone is widely deposited in fat, the fat may act as a depot with large storage capacity, buffering the thyroid gland from excessive exposure to amiodarone. Therefore, it is hypothesized that patients with low BMI have less fat as a buffer, making the thyroid gland more susceptible to the exposure to amiodarone and developing amiodarone‐induced thyroid dysfunction [18]. Although this hypothesis has not been substantiated by measuring amiodarone concentrations in the tissues, it is assumed that the same mechanism explains why APT is more likely to occur in patients with lower BMI.

Patients with APT tended to have a higher prevalence of CKD. In the univariate analysis using the Cox proportional hazards model, both CKD and lower eGFR were statistically significant; however, eGFR did not remain an independent risk factor in the multivariate analysis. Amiodarone and monodesethylamiodarone are primarily eliminated through hepatic metabolism, with only a negligible fraction excreted in the urine [19]. Renal dysfunction, nevertheless, has been linked to a potential reduction in hepatic drug‐metabolizing activity, including the cytochrome P450 3A4 pathway, which plays a central role in amiodarone metabolism [19, 20]. Although some studies have identified CKD as a risk factor for APT [3], others have not demonstrated a consistent association between renal function and the development of APT [4, 13]. Accordingly, the relationship between CKD and APT remains to be fully elucidated.

The plasma concentrations of amiodarone and its metabolites were not identified as significant risk factors for APT onset in logistic regression analysis. Although the maintenance dose of amiodarone emerged as a significant risk factor in the Cox proportional hazards model, this association was not observed in logistic regression. These findings should be interpreted with caution. The Cox proportional hazards model accounts for the time to event, thereby better capturing the increased risk among patients receiving higher doses over a longer duration. In contrast, logistic regression only considers the binary outcome of event occurrence and disregards the time axis, which may lead to an underestimation of cumulative risk and, consequently, a lack of statistical significance.

Although elevated KL‐6 levels have been reported in patients with APT [15], the cutoff value and its sensitivity and specificity of KL‐6 levels during administration as a screening test for APT remain unknown. In our study, the maximum serum KL‐6 value after initiation of amiodarone was greater than the cutoff value of 444 U/mL, with a sensitivity of 70.8% and specificity of 88.1% for diagnosing APT, and the AUC of the ROC curve was relatively high (0.84). Monitoring KL‐6 levels during amiodarone administration may be useful in assessing the background of interstitial lung disease and in suspecting the development of APT.

## 6 Limitations

This study has some limitations. First, this is a single‐centered study with a relatively small number of patients. The small number of APT cases compromises the statistical analysis of potential risk factors, especially when performing analyses on serum KL‐6 levels with even smaller sample sizes. Further population‐based studies with larger sample sizes and prospective trials are warranted to confirm our findings. Second, the cohort was collected at the National Cerebral and Cardiovascular Center, where cardiovascular diseases are mainly treated. This might have caused hospital bias that would select patients with more severe heart failure, younger patients, and patients with fewer non‐cardiovascular comorbidities, such as pulmonary diseases. Although we cannot rule out the possibility that this may have affected the association between pre‐existing lung disease and the development of APT, we believe that selection bias for severe heart failure has little impact on the result because heart failure has not been identified as a risk factor for APT in previous studies.

## 7 Conclusion

In this study, APT occurred in patients who were receiving amiodarone with a maintenance dose of approximately 100 mg, with an incidence of 5.4%. Lower BMI, older age, and higher maintenance dose were risk factors, while plasma concentrations of amiodarone and its metabolites were not significant risk factors. Monitoring of KL‐6 levels may be useful in suspecting the development of APT. Clinicians should consider using the lowest possible dose of amiodarone in elderly, low BMI patients and, if efficacy is not ensured, consider switching to other antiarrhythmic agents or catheter ablation.

## Conflicts of Interest

The authors declare no conflicts of interest.

## Supporting information


**Figure S1:** Flowchart illustrating the analytical process used to assess the accuracy of serum KL‐6 levels as a screening test for APT. APT, amiodarone induced pulmonary toxicity.
**Figure S2:** Receiver‐operating characteristic plot for percent predicted ‐BMI for predicting pulmonary toxicity at the initiation of amiodarone therapy. AUC, area under the curve.
