# Supplementary material for: Low Body Mass Index as a Predictor of Amiodarone‐Induced Pulmonary Toxicity
Source: J Arrhythm. 2025 Oct 22;41(5):e70205. doi: 10.1002/joa3.70205 (PMC12541544; doi:10.1002/joa3.70205)
Supplement: Supplementary file 1 — Figure S1: Flowchart illustrating the analytical process used to assess the accuracy of serum KL‐6 levels as a screening test for APT. APT, amiodarone induced pulmonary toxicity. Figure S2: Receiver‐operating characteristic plot for percent predicted ‐BMI for predicting pulmonary toxicity at the initiation of amiodarone therapy. AUC, area under the curve. [file JOA3-41-e70205-s001.zip › Supplymentary Figure legends.docx]

**Figure legends**

Supplementary Figure 1. Flowchart illustrating the analytical process used to assess the accuracy of serum KL-6 levels as a screening test for APT.

APT, amiodarone induced pulmonary toxicity.

Supplementary Figure 2. Receiver-operating characteristic plot for percent predicted -BMI for predicting pulmonary toxicity at the initiation of amiodarone therapy.

AUC, area under the curve.
