# Supplementary figures and images for: Low Body Mass Index as a Predictor of Amiodarone‐Induced Pulmonary Toxicity
Source: J Arrhythm. 2025 Oct 22;41(5):e70205. doi: 10.1002/joa3.70205 (PMC12541544; doi:10.1002/joa3.70205)

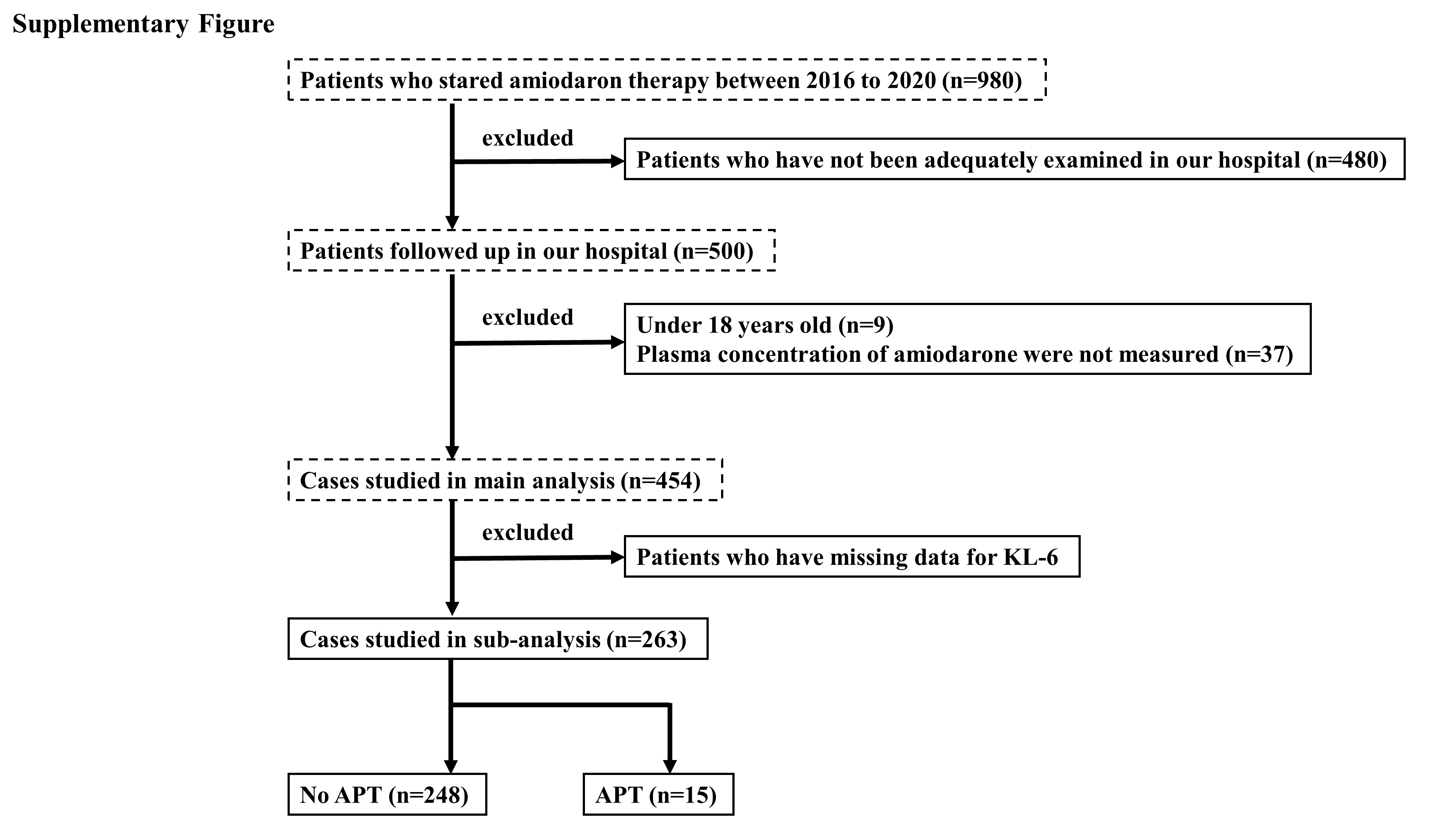

Supplement: Supplementary file 1 — Figure S1: Flowchart illustrating the analytical process used to assess the accuracy of serum KL‐6 levels as a screening test for APT. APT, amiodarone induced pulmonary toxicity. Figure S2: Receiver‐operating characteristic plot for percent predicted ‐BMI for predicting pulmonary toxicity at the initiation of amiodarone therapy. AUC, area under the curve. [file JOA3-41-e70205-s001.zip › joa370205-sup-0001-Supinfo1@Supplemental figure zaizen.tif]

Supplementary Figure 2

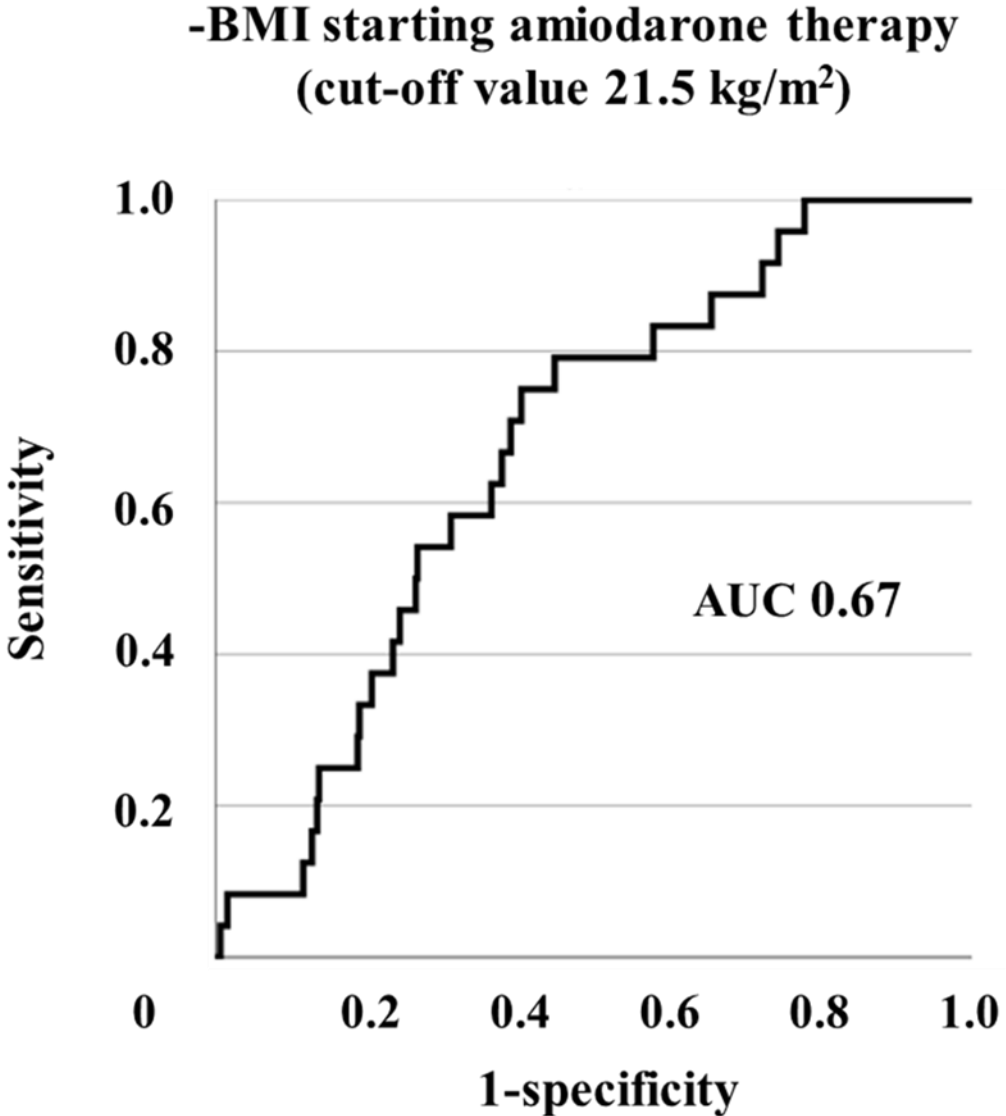

Supplement: Supplementary file 1 — Figure S1: Flowchart illustrating the analytical process used to assess the accuracy of serum KL‐6 levels as a screening test for APT. APT, amiodarone induced pulmonary toxicity. Figure S2: Receiver‐operating characteristic plot for percent predicted ‐BMI for predicting pulmonary toxicity at the initiation of amiodarone therapy. AUC, area under the curve. [file JOA3-41-e70205-s001.zip › joa370205-sup-0002-FigureS2@Supplemental Figure 2.pdf]
